# Diagnostic accuracy, operational feasibility, and cost considerations of Truenat MTB Plus and Xpert MTB/RIF Ultra for pulmonary tuberculosis

**DOI:** 10.1371/journal.pone.0341988

**Published:** 2026-07-08

**Authors:** Zeeshan Sidiq, Ankita Anand, Kaushal Kumar Dwivedi, Payal Tyagi, Sanjay Rajpal, Kamal Kishore Chopra, Shikha Dhawan, Vishal Khanna

**Affiliations:** 1 New Delhi Tuberculosis Centre, JLN Marg, Delhi Gate, New Delhi, Delhi, India; 2 Centre for Social Integration and borderless world, Ghaziabad, Uttar Pradesh, India; 3 Chest Clinic Lok Nayak Hospital, Lok Nayak Jai Prakash Hospital, JLN Marg, Delhi Gate, New Delhi, Delhi, India; Dognosis India Private Limited, INDIA

## Abstract

**Background:**

Tuberculosis (TB) remains a major global health challenge, necessitating rapid and accurate diagnostic tools. The World Health Organization (WHO) endorses nucleic acid amplification tests (NAATs) like Truenat MTB Plus and Xpert MTB/RIF Ultra. While both are widely implemented, direct comparisons integrating diagnostic accuracy with operational and cost considerations across real-world settings are limited. This study aimed to compare the diagnostic accuracy of Truenat MTB Plus and Xpert MTB/RIF Ultra for the detection of Mycobacterium tuberculosis (MTB) and rifampicin resistance (RIF-R) among presumptive pulmonary TB patients in India, while also assessing their operational feasibility and cost-related parameters.

**Methods:**

This prospective diagnostic accuracy study was conducted at New Delhi Tuberculosis Centre, in New Delhi, India, enrolling 664 presumptive pulmonary TB patients. Paired sputum specimens were tested using Truenat MTB Plus/MTB-RIF and Xpert MTB/RIF Ultra. Mycobacterial culture was used as the primary reference standard for MTB detection, and phenotypic drug susceptibility testing (pDST) was used for RIF-R detection. Sensitivity, specificity, and overall accuracy were calculated, and operational parameters, including invalid/indeterminate rates and cost per test, were recorded.

**Results:**

The overall prevalence of culture-confirmed TB was 14.8% (98/664), with a RIF-R prevalence of 7.1% (7/98). Compared with culture, Xpert MTB/RIF Ultra demonstrated a sensitivity of 97.9% (95% CI: 95.2–100%) and a specificity of 95.5% (95% CI: 93.7–97.3%). Truenat MTB Plus showed a sensitivity of 92.5% (95% CI: 85.1–96.6%) and a specificity of 90.1% (95% CI: 86.8–92.6%). For RIF-R detection, Xpert Ultra had a sensitivity of 83.3% and a specificity of 100%, compared to Truenat MTB-RIF’s sensitivity of 75.0% and specificity of 100%.

Operationally, Truenat had a significantly higher rate of invalid results (5.9% vs 0%) and RIF indeterminate results (46.5% vs 13.2%) compared to Xpert Ultra, resulting in an increased need for repeat testing. The cost per test was lower for Truenat (approximately INR 800 for MTB Plus) compared to Xpert MTB/RIF Ultra (approximately INR 1800).

**Conclusion:**

Both Truenat MTB Plus and Xpert MTB/RIF Ultra demonstrated excellent diagnostic accuracy for TB detection. Xpert Ultra showed superior accuracy and lower indeterminate rates, while Truenat offers substantial operational and cost advantages for decentralized deployment; however, higher rates of invalid and indeterminate results may increase operational burden due to repeat testing. These findings support a tiered diagnostic approach, leveraging the strengths of both platforms to expand molecular testing access and accelerate progress toward global TB elimination goals.

## Introduction

Tuberculosis (TB) remains a major global public health challenge, requiring timely and accurate diagnostic tools to enable early treatment and reduce transmission. In 2024, an estimated 10.6 million people developed TB globally, yet only 8.4 million cases were notified, leaving approximately 2.2 million individuals undiagnosed or unreported. Among those diagnosed, a substantial proportion still did not receive upfront testing with a World Health Organization (WHO)–recommended rapid molecular assay and were instead evaluated using less sensitive methods such as smear microscopy [1].

Conventional TB diagnostics have important limitations. Smear microscopy has low sensitivity, particularly in paucibacillary disease, whereas culture, although highly specific, requires prolonged turnaround times. To address these gaps, WHO recommends nucleic acid amplification tests (NAATs) as initial diagnostic tools, as they provide improved sensitivity, rapid results, and the ability to detect drug resistance–associated mutations.

Over the past decade, there has been a global shift toward scaling up molecular diagnostics to achieve universal access to rapid TB testing. In India, under the National Tuberculosis Elimination Programme (NTEP), substantial efforts have been made to expand access to NAATs across both centralized and decentralized healthcare settings. While cartridge-based platforms such as Xpert MTB/RIF Ultra have demonstrated high diagnostic accuracy and ease of use in well-equipped laboratories, their implementation in peripheral settings may be limited by infrastructure requirements, costs, and maintenance needs. In contrast, chip-based real-time PCR platforms, such as Truenat, have been specifically developed for decentralized and near-patient testing, offering advantages in terms of portability, lower infrastructure requirements, and operational flexibility.

Truenat MTB/MTB Plus (Molbio Diagnostics) and Xpert MTB/RIF Ultra (Cepheid) are among the most widely implemented WHO-endorsed NAAT platforms. Truenat is a chip-based, real-time PCR system designed for decentralized testing. The Truenat MTB Plus assay incorporates dual targets (IS6110 and nrdZ) to enhance detection of *Mycobacterium tuberculosis*, particularly in regions where strains with low IS6110 copy numbers are prevalent. Rifampicin resistance detection using the Truenat MTB-RIF assay is based on amplification of the rpoB gene within the rifampicin resistance-determining region (RRDR). In contrast, Xpert MTB/RIF Ultra is a fully automated cartridge-based assay that simultaneously detects *Mycobacterium tuberculosis* (targeting IS6110 and IS1081) and rifampicin resistance through mutations in the rpoB gene, with improved sensitivity in smear-negative and paucibacillary TB, although concerns regarding reduced specificity and infrastructure requirements persist.

Although several studies have evaluated these assays individually, direct head-to-head comparisons that integrate diagnostic accuracy with operational feasibility and cost-related considerations across different healthcare settings remain limited. In this study, operational feasibility was assessed based on parameters including cost per test, infrastructure requirements, turnaround time, throughput, and the frequency of invalid or indeterminate results.

Therefore, this study aimed to compare the diagnostic accuracy of Truenat MTB Plus and Xpert MTB/RIF Ultra among presumptive pulmonary TB patients using mycobacterial culture as the reference standard. Particular emphasis was placed on the head-to-head comparison between the two platforms in a real-world setting, while also assessing their operational feasibility, cost-effectiveness, and scalability.

## Methods

### Study design

This was a prospective cross-sectional diagnostic accuracy study assessing the performance of Xpert MTB/RIF Ultra and Truenat MTB Plus/MTB-RIF at the New Delhi Tuberculosis Centre in New Delhi, India. Participants were consecutively enrolled at presentation, and samples were processed and tested in real time. Participant recruitment for this study was conducted from 04/07/2024–27/12/2024. The study population consisted of patients who presented to the outpatient department of the Chest Clinic, Lok Nayak Jaiprakash Hospital, with a clinical suspicion of pulmonary tuberculosis. Participants were consecutively enrolled if they fulfilled the following criteria: (i) provided written informed consent, (ii) had no prior history of anti-tuberculosis therapy, and (iii) were able to submit adequate quality sputum specimens.

The study aimed to recruit approximately 1000 participants to obtain at least 100 *Mycobacterium tuberculosis* (MTB)–positive specimens and a minimum of 20 rifampicin-resistant (RIF-R) specimens. The sample size was calculated with α = 0.05 and 80% power based on reported sensitivities of 0.88 for Xpert MTB/RIF Ultra and 0.73 for Truenat MTB Dx [2]. Enrollment was concluded once the predefined recruitment targets had been achieved. This study adhered to the ethical principles outlined in the Declaration of Helsinki (1964) and its subsequent revisions. Approval was obtained from the appropriate institutional scientific and ethics committees.

### Procedures

The enrolled participants were requested to provide two consecutive sputum specimens, which were promptly transported to the laboratory. The paired samples were pooled, homogenized, and divided into three equal aliquots. Operational feasibility was assessed using predefined parameters as described above. One aliquot was tested using the Xpert MTB/RIF Ultra assay (Cepheid, Sunnyvale, CA, USA; Catalog No: GXMTB-ULTRA-MII-10), while the second with the Truenat MTB Plus assay (Molbio Diagnostics Pvt Ltd, Goa, India; Catalog No: 601130005). The Truenat platform follows a sequential two-step workflow, wherein MTB detection is performed using Truenat MTB Plus, followed by rifampicin resistance testing using Truenat MTB-RIF only in MTB-positive samples. In contrast, Xpert MTB/RIF Ultra performs simultaneous detection of *Mycobacterium tuberculosis* and rifampicin resistance in a single cartridge.

The third aliquot was used for smear microscopy and mycobacterial culture. Smear microscopy was performed using fluorescence microscopy (Auramine-O staining), and mycobacterial culture was performed using the BACTEC MGIT 960 system (BD Diagnostics, USA). Pooling was performed to ensure that all diagnostic tests were conducted on identical material, thereby enabling a direct and reliable comparison of assay performance. All procedures for smear microscopy, liquid culture, Drug Susceptibility testing (DST), Truenat, and GeneXpert were performed following standard protocols [3–7].

Liquid culture was used as the primary reference standard for evaluating the diagnostic accuracy of Truenat MTB Plus and Xpert MTB/RIF Ultra, in line with WHO recommendations. Sensitivity, specificity, positive predictive value (PPV), negative predictive value (NPV), and overall accuracy were calculated using culture-positive and culture-negative specimens. A composite reference standard (CRS)—defined as positivity by either liquid culture or smear microscopy—was applied as a secondary analysis to account for potential false-negative culture results; CRS-based estimates were considered supportive. Phenotypic drug susceptibility testing (pDST) on culture-positive isolates served as the reference standard for rifampicin resistance detection. Indeterminate rifampicin resistance results were excluded from accuracy calculations and analyzed separately for operational implications. 95% confidence intervals were calculated using the Wilson method, and rifampicin resistance analyses were considered exploratory due to the limited number of resistant cases.

*Note: Diagnostic accuracy analyses were performed using only samples for which valid results were available for both the index test and the corresponding reference standard. Consequently, denominators varied across comparisons due to invalid molecular test results, contaminated cultures, or unavailability of phenotypic drug susceptibility testing.*

## Results

During the study period, a total of 664 participants were included in the study, comprising 382 males (57.5%) and 282 females (42.5%), with a male-to-female ratio of approximately 1.35:1. The participants’ ages ranged from 10 to 91 years, with a median age of 36.5 years. The largest representation was from the 36–59 years group (240/664, 36.1%), followed closely by the 19–35 years group (217/664, 32.7%). Overall, the cohort was predominantly adult, with substantial representation of both pediatric and elderly populations, allowing for a robust demographic analysis. A total of 98 patients were culture-confirmed for *Mycobacterium tuberculosis*, giving an overall Tuberculosis prevalence of 14.8%. Rifampicin resistance was detected in seven cases, corresponding to an overall prevalence of 7.1%. The detailed results are given in Table 1.

**Table 1:** Age- and Sex-wise Distribution of TB and Rifampicin Resistance.

Age Group	Male (n)	Female (n)	Total (n)	% of total	Culture-positive TB (n)	TB prevalence (%)	Rifampicin-resistant TB (n)	RR-TB prevalence (%)
≤18	51	51	102	15.4	17	16.7	1	5.9
19–35	107	110	217	32.7	35	16.1	2	5.7
36–59	145	95	240	36.1	35	14.6	3	8.6
≥60	79	26	105	15.8	11	10.5	1	9.1
**Total**	**382**	**282**	**664**	**100**	**98**	**14.7**	**7**	**7.1**

### Diagnostic accuracy of truenat MTB plus assay

**Fig 1:**
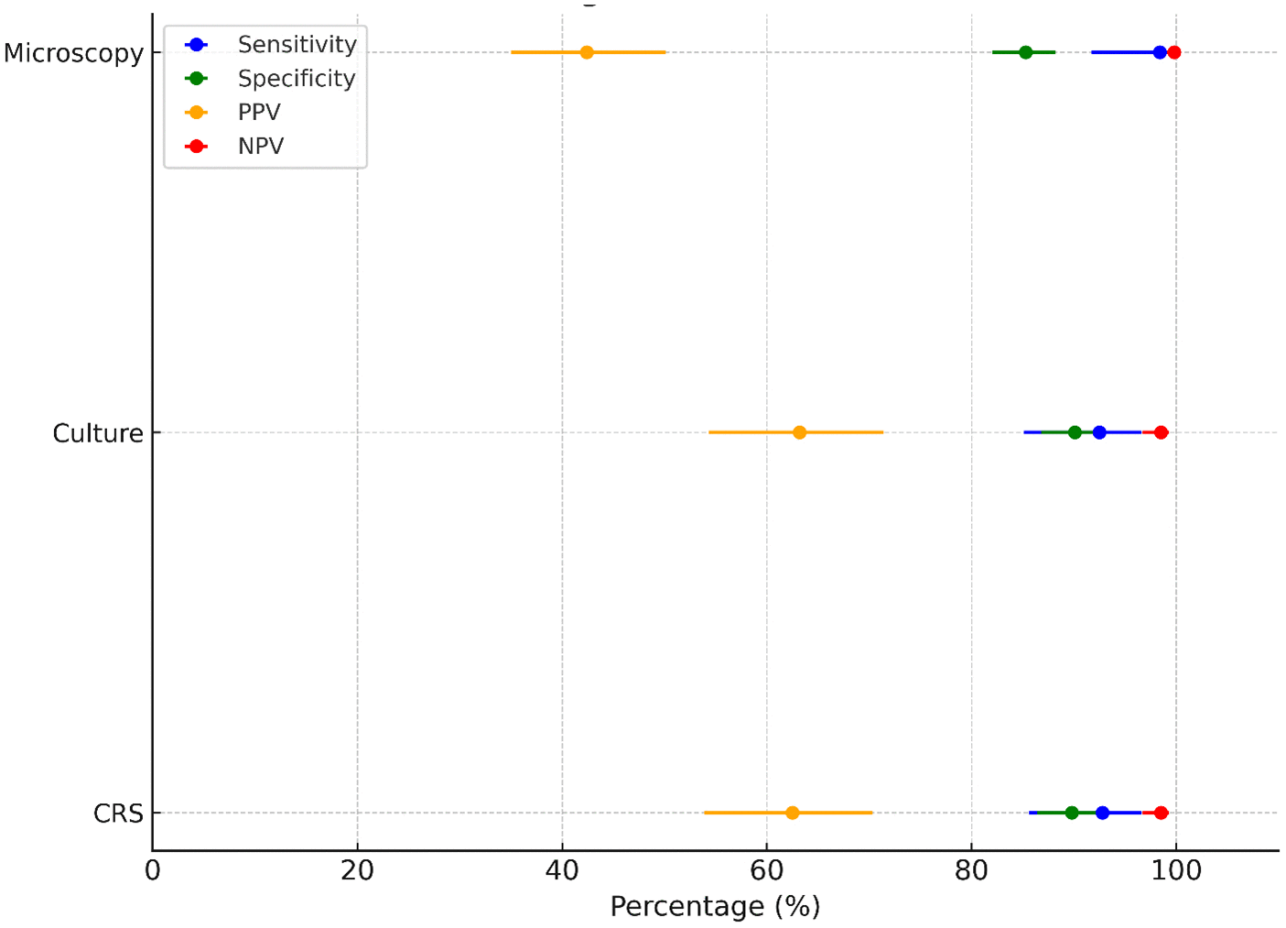
Forest Plot for the diagnostic accuracy of Truenat MTB Plus.

Truenat MTB Plus demonstrated a sensitivity of 98.4% (95% CI: 91.7–99.9%) and specificity of 85.3% (95% CI: 82.0–88.2%) against microscopy (N = 625), with a PPV of 42.4%, NPV of 99.8%, and an overall accuracy of 86.0%. Compared with culture (N = 596), the sensitivity and specificity were 92.5% (85.1–96.6%) and 90.1% (86.8–92.6%), respectively, with PPV 63.2%, NPV 98.5%, and accuracy 90.4%. Against a composite reference standard (smear or culture; N = 625), MTB Plus achieved 92.8% sensitivity (85.6–96.6%), 89.8% specificity (86.4–92.4%), PPV 62.5%, NPV 98.5%, and an accuracy of 90.2%. For rifampicin resistance detection, MTB-RIF compared with phenotypic DST (N = 64) showed 75.0% sensitivity (30.1–95.4%), 100% specificity (93.9–100%), PPV 100%, NPV 98.4%, and an overall accuracy of 98.4%. The results are presented in Table 2 and Fig 1.

**Table 2:** Diagnostic performance of Truenat MTB Plus and Truenat MTB-RIF Dx compared with microscopy, culture, and composite reference standard and pDST for tuberculosis and rifampicin resistance detection.

	N	True Positive	True Negative	False Positive	False Negative	Sensitivity (95% CI)	Specificity (95% CI)	PPV (95% CI)	NPV (95% CI)	Accuracy
MTB plus vs Microscopy	625	61	480	83	1	98.4% (91.7–99.9)	85.3% (82.0–88.2)	42.4% (35.0–50.1)	99.8% (98.7–100)	86.0%
MTB plus vs Culture	596	86	453	50	7	92.5% (85.1–96.6)	90.1% (86.8–92.6)	63.2% (54.3–71.4)	98.5% (96.7–99.3)	90.4%
MTB Plus vs CRS (smear or culture)	625	90	474	54	7	92.8% (85.6–96.6)	89.8% (86.4–92.4)	62.5% (53.9–70.3)	98.5% (96.7–99.3)	90.2%
MTB-RIF vs pDST	64	3	60	0	1	75.0% (30.1–95.4)	100% (93.9–100)	100% (43.9–100)	98.4% (91.3–99.7)	98.4%

CRS- Composite Reference Standard; pDST- Phenotypic drug susceptibility testing.

### Diagnostic accuracy of the Xpert MTB/RIF ultra assay

**Fig 2:**
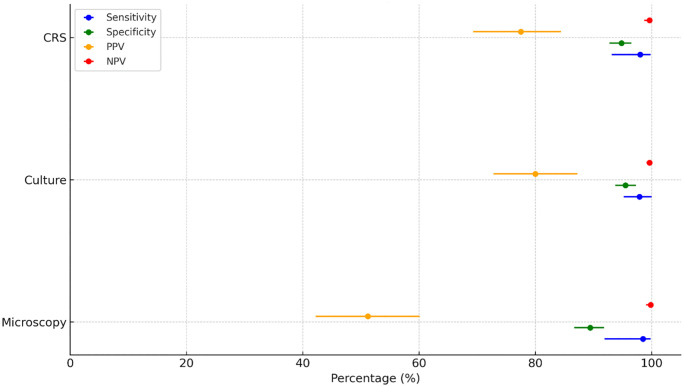
Forest Plot for the diagnostic accuracy of Xpert MTB/RIF Ultra. Comparison of errors, invalid, and indeterminate results between Truenat MTB Plus and Xpert MTB/RIF Ultra.

Against microscopy (N = 663), Xpert MTB/RIF Ultra showed a sensitivity of 98.5% (95% CI: 91.9–99.8%) and specificity of 89.4% (95% CI: 86.7–91.8%), with a PPV of 51.2% and NPV of 99.8%, yielding an accuracy of 90.3%. Compared with culture (N = 633), the sensitivity and specificity were 97.9% (95% CI: 95.2–100%) and 95.5% (95% CI: 93.7–97.3%), respectively, with an accuracy of 95.9%. Against the composite reference standard (smear or culture, N = 663), the sensitivity was 98.0% (95% CI: 93.1–99.8%) and specificity was 94.8% (95% CI: 92.7–96.5%), achieving 95.3% accuracy (Fig 2). Xpert MTB/RIF Ultra versus phenotypic DST (N = 93) for rifampicin resistance detected showed 83.3% sensitivity (95% CI: 43.6–97.0%) and 100% specificity (95% CI: 95.8–100%), with an accuracy of 98.9%. A direct comparison between Truenat MTB Plus and Xpert MTB/RIF Ultra is presented in Table 3.

**Table 3:** Comparative performance of Truenat MTB Plus and Xpert MTB/RIF Ultra.

	N	True Positive	True Negative	False Positive	False Negative	Sensitivity (95% CI)	Specificity (95% CI)	PPV (95% CI)	NPV (95% CI)	Accuracy
Xpert vs Microscopy	663	66	533	63	1	98.5% (91.9–99.8)	89.4% (86.7–91.8)	51.2.4% (42.2–60.1)	99.8% (99.0–100)	90.3%
Xpert vs Culture	633	96	511	24	2	97.9% (95.2–100)	95.5% (93.7–97.3)	80.0% (72.8–87.2)	99.6% (99.1–100)	95.9%
Xpert vs CRS (smear or culture)	663	100	532	29	2	98.0% (93.1–99.8)	94.8% (92.7–96.5)	77.5% (69.3–84.4)	99.6% (98.7–99.9)	95.3%
RIF Ultra vs pDST	93	5	87	0	1	83.3% (43.6–97.0)	100% (95.8–100)	100% (56.6–100)	98.9% (93.8–99.8)	98.9%

CRS- Composite Reference Standard; pDST- Phenotypic drug susceptibility testing.

### Sample processing and extraction-related Issues

During sample processing, occasional challenges were noted. Some samples failed to liquefy within the stipulated time despite extended or overnight incubation, while 74 instances of cartridge clogging were observed during DNA extraction with the Trueprep system. Though infrequent, these events affected workflow efficiency and sample quality. In contrast, with the Xpert MTB/RIF Ultra assay, only two errors (codes 5006 and 5011) were observed.

### Detection errors in MTB assays

A total of 39 (5.9%) invalid results were obtained for *Mycobacterium tuberculosis* detection using the Truenat MTB Plus assay, whereas no invalid results were observed with Xpert MTB/RIF Ultra. Following a single repeat test, valid results were obtained in 3 of 39 initially invalid samples, corresponding to a reduction of 7.7% in invalid results, while 36 samples (92.3%) remained invalid.

### Indeterminate results for rifampicin resistance detection

Among the samples that tested positive for *Mycobacterium tuberculosis,* 46.5% (67 out of 144) from the Truenat MTB Plus assay yielded indeterminate results for rifampicin resistance when tested with Truenat MTB-RIF. In comparison, 17 out of 129 (13.2%) MTB-positive samples tested by Xpert MTB/RIF Ultra were indeterminate for rifampicin resistance. Notably, all 17 indeterminate results on Xpert MTB/RIF Ultra corresponded to trace-positive MTB detections, whereas the Truenat MTB Plus assay showed indeterminate outcomes across a range of MTB detection grades, from very low to high.

Since indeterminate rifampicin resistance results associated with trace-positive MTB detections are inherent to the assay design, these were analyzed separately. Excluding trace-positive results, no indeterminate rifampicin resistance results were observed with Xpert MTB/RIF Ultra.

## Operational feasibility and cost-related parameters

Operational and cost-related parameters were evaluated to assess assay feasibility across different healthcare settings. Key factors included cost per test, infrastructure requirements, turnaround time, and the frequency of invalid or indeterminate results. Data on assay throughput, infrastructure needs, and cost per test were recorded to support the subsequent analysis of cost-effectiveness and scalability. In addition, the need for repeat testing in cases of invalid and indeterminate results was considered as a contributor to overall operational cost and laboratory workload. The results are detailed in Table 4.

**Table 4:** Comparison of Operational and Cost-related Parameters Between Truenat MTB Plus / RIF and Xpert MTB/RIF Ultra.

Parameter	Truenat MTB Plus / RIF	Xpert MTB/RIF Ultra
Cost per test (approx., INR)	800 + 562*	1800
Instrument cost	Low–Moderate	High
MTB invalid results	39/625 (6.2%)	0/625 (0%)
RIF indeterminate results	67/144 (46.5%)	17/129 (13.2%)**
Cartridge clogging	74 instances	0
Instrument errors	13	2 (5006, 5011)
Repeat testing required	52/625 (8.3%)	2/625 (0.3%)
Average turnaround time	60–90 min/sample	~90 min/sample
Throughput (samples/run)	1–4	1–4
Infrastructure needs	Minimal	Moderate–High
Suitable level of use	Peripheral / District	District / Reference

*800 for MTB Plus, and additional 562 for RIF chip; ** All indeterminate results in Xpert MTB/RIF Ultra were associated with trace-positive MTB detections and were excluded from comparative analysis.

## Discussion

This study offers a detailed, real-world comparison of two WHO-endorsed molecular diagnostic tools, Truenat MTB Plus and Xpert MTB/RIF Ultra, for the detection of *Mycobacterium tuberculosis* and rifampicin resistance among individuals with presumptive pulmonary TB in India. Both platforms performed well overall; however, important distinctions in accuracy, operational reliability, and error rates were observed that are relevant to national TB control efforts and the global drive for universal access to rapid drug-resistant testing. The primary strength of this study lies in the head-to-head comparison of Truenat MTB Plus and Xpert MTB/RIF Ultra under routine programmatic conditions.

Using culture as the reference standard, Xpert MTB/RIF Ultra achieved slightly higher sensitivity and specificity (97.9% and 95.5%, respectively) than Truenat MTB Plus (92.5% and 90.1%, respectively). This modest but consistent advantage mirrors the results of earlier multicentric evaluations that reported similar findings in Indian and African settings [8–10]. The improved analytical sensitivity of Xpert MTB/RIF Ultra likely stems from its larger reaction volume, refined probe chemistry, and algorithm optimized for low bacillary load samples.

Both assays clearly outperformed smear microscopy, confirming the superiority of molecular testing for diagnosis of TB. Several studies have demonstrated that molecular assays have higher sensitivity than smear microscopy, particularly in smear-negative and low bacillary load cases [10,11]. Their high negative predictive values (≥98%) indicate that either test can reliably exclude disease in symptomatic individuals, which is an important consideration for screening and triage in high-burden programs.

Reliable detection of rifampicin resistance is essential to ensure that patients receive effective therapy early. In this evaluation, both assays demonstrated perfect specificity compared with phenotypic drug susceptibility testing, confirming that false-positive resistance calls were rare. However, Xpert MTB/RIF Ultra showed higher sensitivity (83.3%) than Truenat MTB-RIF (75.0%). Due to the limited number of rifampicin-resistant cases, these sensitivity estimates should be interpreted with caution. The small number of rifampicin-resistant cases also resulted in wider confidence intervals, limiting the precision of comparative estimates between the two platforms.Given the limited number of rifampicin-resistant cases, agreement-based analysis was considered; however, sensitivity and specificity were retained to maintain consistency with the standard diagnostic accuracy reporting. Xpert MTB/RIF Ultra also demonstrated a markedly lower proportion of indeterminate results (13.2% vs. 46.5%). Indeterminate rifampicin resistance results necessitate repeat testing, referral to higher-tier laboratories, or delayed treatment decisions, all of which can impact patient management and laboratory workload. All Xpert MTB/RIF Ultra-indeterminate results occurred in samples with trace-positive MTB detections, consistent with the algorithmic safeguards to prevent spurious resistance reporting in very low bacterial loads [5]. These results were therefore analyzed separately and excluded from direct comparison with Truenat MTB-RIF, as rifampicin indeterminate results in trace-positive samples are an inherent feature of the assay and reflect low bacillary load rather than assay failure. In contrast, Truenat produced indeterminate outcomes across all detection categories, suggesting variability related to amplification or internal control thresholds. Comparable findings have been described in previous Truenat evaluations [7,8,12,13], where the multi-step extraction plus amplification workflow may have contributed to reduced assay stability. In contrast, Xpert MTB/RIF Ultra’s integrated closed-cartridge design limits operator handling and potential DNA loss, which likely explains its greater consistency across sample types [7,14,15].

From a programmatic standpoint, both technologies offer clear advantages but serve different operational needs. Truenat, with its small footprint and portability, is well-suited for decentralized or peripheral deployment, allowing same-day results and faster treatment initiation in hard-to-reach areas. Xpert MTB/RIF Ultra, which offers superior analytical precision and minimal manual intervention, is best positioned for district or reference laboratories where confirmatory testing, surveillance, and quality monitoring are conducted. This complementary approach aligns with the WHO’s current guidance that molecular platforms should be deployed according to local infrastructure and case load to maximize access and maintain high diagnostic standards [6]. Therefore, a network that integrates both systems could ensure wider diagnostic coverage while preserving analytical rigor.

In addition to the per-test costs, operational factors such as MTB invalid, indeterminate, and rifampicin indeterminate results contribute significantly to the overall programmatic costs and laboratory workload. These outcomes necessitate repeat testing, increased consumable use, and, in some cases, referral to higher-tier laboratories, thereby increasing the turnaround time and resource utilization. In this study, the higher proportion of such results observed with Truenat MTB Plus and MTB-RIF suggests an increased operational burden despite the lower upfront test costs.

The main strengths of this investigation are its prospective design, use of identical pooled sputum for both assays, eliminating inter-sample variability, and adherence to standardized WHO and Global Laboratory Initiative procedures. These factors increase the comparability with other datasets and enhance confidence in the findings.

This study has certain limitations. The number of rifampicin-resistant cases identified during the study period was limited, and therefore estimates related to rifampicin resistance detection should be interpreted with caution. The study was conducted at a single high-volume diagnostic centre, which may limit generalizability to settings with lower testing throughput. Indeterminate results were excluded from diagnostic accuracy calculations; however, these were analyzed separately to highlight their operational implications. This study included pulmonary tuberculosis cases; extrapulmonary samples were not evaluated. Although both smear-positive and smear-negative cases were included, subgroup analysis based on smear status was not performed, which may influence interpretation of assay performance in low bacillary load specimens [10,16]. Finally, the cost analysis was descriptive in nature and intended to provide comparative insights rather than a formal economic evaluation.

In conclusion, both Truenat MTB Plus and Xpert MTB/RIF Ultra demonstrated excellent diagnostic accuracy for detecting *Mycobacterium tuberculosis* and rifampicin resistance. Xpert MTB/RIF Ultra showed higher sensitivity, specificity, and reproducibility, particularly for rifampicin resistance detection, with significantly fewer indeterminate results. Truenat MTB Plus, while slightly less precise, offers clear operational advantages through its portability, lower cost, and minimal infrastructure requirements, making it highly suitable for decentralized testing. Together, these findings support a tiered diagnostic approach—deploying Truenat to expand molecular testing at peripheral and district levels and Xpert MTB/RIF Ultra at higher-tier laboratories for confirmatory testing—to strengthen diagnostic networks, improve access to rapid drug-resistance testing, and advance progress toward global TB elimination goals.
