# Supplementary material for: Diagnostic accuracy, operational feasibility, and cost considerations of Truenat MTB Plus and Xpert MTB/RIF Ultra for pulmonary tuberculosis
Source: PLoS One. 2026 Jul 8;21(7):e0341988. doi: 10.1371/journal.pone.0341988 (PMC13345227; doi:10.1371/journal.pone.0341988)
Supplement: Attachment — Submitted filename: report_10.64898+2026.01.19.26344389.pdf [file pone.0341988.s001.pdf]

ScreenIT Report

This is an automated report for 10.64898+2026.01.19.26344389.pdf. This report was solicited by the PLOS One editorial team and provided by ScreenIT.

ScreenIT is an independent group of scientists developing automated tools that analyze academic papers. A set of automated tools screened your submitted manuscript and provided the report below. Each tool was created by your academic colleagues with the goal of helping authors.

The tools look for factors that are important for transparency, rigor and reproducibility, and we hope that the report might help you to improve reporting in your manuscript. Within the report you will find links to more information about the items that the tools check. These links include helpful papers, websites, or videos that explain why the item is important.

While our screening tools aim to improve and maintain quality standards they may, on occasion, miss nuances specific to your study type or flag something incorrectly. Each tool has limitations that are described on the ScreenIT website. The tools screen the main file for the paper; they are not able to screen supplements stored in separate files.

Please note that the Academic Editor had access to these comments while making a decision on your manuscript. The Academic Editor may ask that issues flagged in this report be addressed.

If you would like to provide feedback on the ScreenIT tool, please email the team at ScreenIt@bih-charite.de. If you have questions or concerns about the review process, please contact the PLOS One office at plosone@plos.org.

How to read the report:

- no action required
- review for potential issues
- a critical issue was found by a tool
- may not be applicable to your paper

- more information about this item can be obtained using this link

Rigor

✓	Ethics	Consent: "Participants were consecutively enrolled if they fulfilled the following criteria: (i) provided written informed consent, (ii) had no prior history of anti-tuberculosis therapy, and (iii) were able to submit adequate quality sputum specimens."<br>IRB: "Approval was obtained from the appropriate institutional scientific and ethics committees."
✓	Inclusion and Exclusion Criteria	"Procedures: The enrolled participants were requested to provide two consecutive sputum specimens, which were promptly transported to the laboratory."
?	Flow charts and attrition	We did not find a study flow chart of excluded observations. We strongly recommend using flow charts because they provide an overview of the study design and more information about attrition. If you included a study flow chart in your supplemental files, we apologize for missing this. Our tool is not able to screen separate supplemental files.<br><br>Sentence about attrition: not detected. Please provide information about the drop-out of subjects, or loss of animals or samples. This could be done using a study flow chart, or described in the text.
✓	Sex as a biological variable	"Results During the study period, a total of 664 participants were included in the study, comprising 382 males (57.5%) and 282 females (42.5%), with a male-to-female ratio of approximately 1.35:1."
✓	Subject Demographics	Age: "The participants ages ranged from 10 to 91 years, with a median age of 36.5 years."
?	Randomization	Not detected. If you performed an experimental study, please specify whether participants, animals or samples were randomly assigned to treatments or groups, and specify the randomization procedure.
	Blinding	Not detected. Please specify whether blinding was used at various phases of the

?		experiment (e.g., blinding of patients, caregivers, outcome assessments, data analysis).
✓	Power Analysis	"The sample size was calculated with = 0.05 and 80% power based on reported sensitivities of 0.88 for Xpert MTB/RIF Ultra and 0.73 for Truenat MTB Dx (2)."
n/a	Replication	not required.

Generated by Barzooka (flow charts), SciScore (everything else).
SciScore only screens the text of the methods section; we apologize if the tool has missed items reported elsewhere in the paper or supplements.

Resources

Persistent unique identifiers for research resources, RRIDs, can be used to track these key resources though the literature. They are requested or required in many journals, but even if your journal does not require them, we strongly encourage the use of these for the readers of papers who wish to easily track down the key materials like antibodies, software tools, or mice.

Recombinant DNA	"Table 2: Diagnostic performance of Truenat MTB Plus and Truenat MTB-RIF Dx compared with microscopy, culture, and composite reference standard and pDST for tuberculosis and rifampicin resistance detection."	? pDST suggested: None. Please add an RRID to help others identify this resource.

Generated by SciScore.
SciScore only screens the text of the methods section; we apologize if the tool has missed items reported elsewhere in the paper or supplements.

Transparency

?	Open code	If you wrote code to analyze your data, please consider sharing the code in a public

		repository. This makes it easier for others to reproduce your analyses, and may also aid others seeking to analyze similar datasets.
?	Open data	ℹ If permitted, sharing data on a public repository with a persistent identifier (a DOI or accession number) can improve reproducibility and make it easier for other scientists to expand on your work. Papers with open data are cited more often than papers without open data. Some institutions have an expert who can provide advice on data sharing.
?	Self-acknowledged limitations	ℹ An explicit section about the limitations of this study was not found. We encourage authors to include a paragraph in the discussion that addresses study limitations. Every study has limitations. Describing these limitations helps readers to understand and contextualize the research.
?	Conflict of interest statement	ℹ It is important to supply a conflict of interest statement, even if there are none to declare, to transparently present the research.
?	Funding statement	ℹ It is important to supply a funding statement because it makes the funding source and potential conflicts of interests transparent.
n/a	Registration statement	ℹ A registered protocol makes the planned research project more transparent and can protect against bias. Please consider registering future confirmatory studies.

Generated by ODDPub, limitation-recognizer, rtransparent, trial-identifier.

Figures

✓	No bar graphs of continuous data	ℹ Thank you for not using bar graphs to present continuous data. Many different datasets and data distributions can lead to the same bar graph, and the actual data may suggest different conclusions from the summary statistics alone.
✓	No rainbow color maps	ℹ Thank you for not using rainbow colormaps. Rainbow color maps distort readers’ perception of the data by introducing visual artifacts and are not accessible to colorblind readers.

Generated by JetFighter, Barzooka.
