# Supplementary material for: Diagnostic accuracy, operational feasibility, and cost considerations of Truenat MTB Plus and Xpert MTB/RIF Ultra for pulmonary tuberculosis
Source: PLoS One. 2026 Jul 8;21(7):e0341988. doi: 10.1371/journal.pone.0341988 (PMC13345227; doi:10.1371/journal.pone.0341988)
Supplement: Attachment — Submitted filename: Response to Reviewers.docx [file pone.0341988.s002.docx]

**Academic Reviewer**

**Comment 1: The introduction could be expanded with additional background on molecular diagnostics for TB and supported with relevant references.**

**Response:** We thank the reviewer for this suggestion. Additional background on molecular diagnostics for tuberculosis has been incorporated into the Introduction section to better contextualize the role of rapid molecular assays in TB diagnosis.

**Comment 2*:* Please provide the manufacturer details and catalog numbers of the kits used for both assays.**

**Response:** We thank the reviewer for this suggestion. Manufacturer details and catalog numbers for both Xpert MTB/RIF Ultra and Truenat MTB Plus/MTB-RIF assays have been added in the Methods (Procedures) section.

**Comment 3: The rifampicin resistance targets for the TruPCR assay are not described in the introduction and should be included.**

**Response:** We thank the reviewer for this observation. The targets for rifampicin resistance detection in the Truenat MTB-RIF assay have now been described in the Introduction section.

**Comment 4: Additional methodological clarity on both platforms would be helpful. Please clarify whether the assay follows a two-step approach and gene targets.**

**Response:** We thank the reviewer for this valuable suggestion. The Methods section has been revised to provide additional clarity on the workflow of both platforms. The sequential two-step process for Truenat (MTB detection followed by MTB-RIF testing) and the single-step integrated workflow for Xpert MTB/RIF Ultra have been clearly described.

**Comment 5: Minor editorial correction required in line 235 (‘TB diagnosis of TB’ – repetition). Supporting references comparing molecular assays with smear microscopy would strengthen this statement.**

**Response:** We thank the reviewer for identifying this error. The typographical repetition has been corrected. Additionally, the statement has been strengthened by including supporting literature highlighting the superior sensitivity of molecular assays compared to smear microscopy, particularly in smear-negative cases.

**Comment 6: In the limitations section, clarify whether smear-negative or extrapulmonary samples were included**

**Response:** We thank the reviewer for this important suggestion. The limitations section has been revised to clarify that only pulmonary TB cases were included. It has also been specified that, although both smear-positive and smear-negative cases were included, subgroup analysis based on smear status was not performed.

**Reviewer# 1**

**Comment1: The author needs to define feasibility from the beginning. Which parameters were used to measure feasibility?**

**Response:** We thank the reviewer for this suggestion. Operational feasibility has now been clearly defined in the Introduction section, including the parameters used for its assessment.

**Comment 2:  L52-54: This is discussing results, it should be reserved to the conclusion section**

**Response:** We thank the reviewer for this observation. Result statements have been retained in the Results section in a descriptive manner, and interpretative statements have been moved

**Comment 3: L57: There are no results for smear microscopy, yet it makes it to the conclusion section**

**Response:** We thank the reviewer for this observation. The comparison with smear microscopy has been removed from the conclusion section of the abstract to ensure consistency with the results presented.

**Comment 4: L78: please use 2025 WHO TB report for 2024 data**

**Response:** We thank the reviewer for this suggestion. The epidemiological data in the Introduction have been updated in accordance with the latest *WHO Global Tuberculosis Report 2025*, reflecting the most recent global TB burden estimates.

**Comment 5: 106: The prospective nature of the study design, is not clearly spelt out: how it will be done and for which part of the design**

**Response:** The study design has been clarified as a prospective cross-sectional diagnostic accuracy study, and details regarding consecutive enrollment and real-time sample processing have been explicitly described.

**Comment 6: Results L147-149: This is repeating the methods under results section. Please revise to reduce on the wording.**

**Response:** We thank the reviewer for this observation. The Results section has been revised to remove redundant methodological descriptions and streamline the wording to present only key findings.

**Comment 7:  Was smear microscopy using ZN or fluorescent Microscopy? please clarify**

**Response:** We thank the reviewer for this suggestion. The methods used for smear microscopy and mycobacterial culture have now been clearly specified in the Methods section, including the use of fluorescence microscopy (Auramine-O staining) and the BACTEC MGIT 960 system for culture.

**Comment 8: L166-167: The sensitivity for DST using phenotypic DST as reference had few samples, why not use agreement instead of sensitivity since this has less power as depicted by the wider confidence intervals. Alternatively compare agreement for DST between TrueNAT and GeneXpert and use phenotypic DST as a tie-breaker.**

**Response:** We thank the reviewer for this insightful suggestion. We acknowledge that the limited number of rifampicin-resistant cases affects the precision of sensitivity estimates, as reflected by wider confidence intervals. While agreement-based analysis was considered, sensitivity and specificity measures were retained to ensure consistency with standard diagnostic accuracy reporting. This limitation has now been explicitly discussed in the manuscript.

**Comment 9: Please review the PPV for MTB plus vs Microscopy: I get 53.5% instead of 42.4%**

**Response:** We thank the reviewer for carefully examining the data. We have rechecked the PPV calculation for MTB Plus versus microscopy. Based on the values provided in Table 2 (TP = 61, FP = 83), the PPV is 61/(61+83) = 42.4%, which is correctly reported in the manuscript. We have reconfirmed that all values in the table are internally consistent.

**Comment 10:  MTB plus vs Culture and MTB-RIF Vs pDST has been done before, what is new that this data is providing? This manuscript is focused on Mtbplus vs Xpert which should be the main focus. The same with Table 3.**

**Response:** We thank the reviewer for this important observation. The Discussion has been revised to emphasize the head-to-head comparison between Truenat MTB Plus and Xpert MTB/RIF Ultra as the primary focus of the study. This has been explicitly highlighted in the opening paragraph of the Discussion.

**Comment 11:  Errors and invalids should indicate that it is comparing MTBplus and Xpert in the sub title**

**Response:** We thank the reviewer for this suggestion. The subheading has been revised to clearly indicate that the comparison is between Truenat MTB Plus and Xpert MTB/RIF Ultra.

**Comment 12: L201-202: please report in terms of the reduction in invalid results**

**Response:** We thank the reviewer for this suggestion. The reduction in invalid results following repeat testing has now been explicitly quantified in the Results section.

**Comment 13: The Rif indeterminate for TRACE call under Xpert, is expected as per the technology, why not present it separate without comparison? Compare Inditerminate and errors for RIF between platforms without TRACE call results.**

**Response:** We thank the reviewer for this important observation. Indeterminate rifampicin resistance results in Xpert MTB/RIF Ultra were found to occur exclusively in trace-positive samples, which are an inherent, algorithm-defined feature of the assay. These have now been presented separately and excluded from direct comparison with Truenat MTB-RIF. The Results, Discussion, and corresponding table have been revised accordingly for clarity.

**Comment 14:  Table 4 (which is referred as Table 3): MTB invalid, indeterminate and RIF indeterminate, ALL contribute to cost and must be discussed. They also require repeat testing and should be discussed under cost and feasibility section.**

**Response:** We thank the reviewer for this important observation. The Discussion section has been revised to include the impact of MTB invalid, indeterminate, and rifampicin indeterminate results on cost and operational feasibility, including the need for repeat testing and increased resource utilization.
