# Supplementary material for: Diagnostic accuracy, operational feasibility, and cost considerations of Truenat MTB Plus and Xpert MTB/RIF Ultra for pulmonary tuberculosis
Source: PLoS One. 2026 Jul 8;21(7):e0341988. doi: 10.1371/journal.pone.0341988 (PMC13345227; doi:10.1371/journal.pone.0341988)
Supplement: Peer review history

# Peer review: Decision Letter 0

**DOI:** 10.1371/journal.pone.0341988.r001

**Authors:** Minal Dakhave

10 Mar 2026

PONE-D-26-00956

Diagnostic accuracy, operational feasibility, and cost considerations of Truenat MTB Plus and Xpert MTB/RIF Ultra for pulmonary tuberculosis

PLOS One

Dear Dr. Sidiq

Thank you for submitting your manuscript to PLOS ONE. After careful consideration, we feel that it has merit but does not fully meet PLOS ONE’s publication criteria as it currently stands. Therefore, we invite you to submit a revised version of the manuscript that addresses the points raised during the review process.

==============================

**Overall Assessment:**

The manuscript addresses an important topic in tuberculosis diagnostics and provides useful comparative data between two WHO-recommended molecular platforms. With minor clarifications and additional methodological details, the study could provide valuable insights for diagnostic laboratories and TB control programs.

**Decision:**  Minor Revision

**Recommendations:**

1. The **introduction could be expanded**  with additional background on molecular diagnostics for TB and supported with relevant references.
2. Please provide the **manufacturer details and catalog numbers**  of the kits used for both assays.
3. The **rifampicin resistance targets for the TruPCR assay**  are not described in the introduction and should be included.
4. Additional methodological clarity on both platforms would be helpful. Please clarify whether the assay follows a **two-step approach**  (initial MTB detection followed by a second PCR for RIF resistance if MTB is positive). For **GeneXpert** , the specific **gene targets used for MTB detection and rifampicin resistance**  should also be described.
5. Minor editorial correction required in line 235 (“TB diagnosis of TB” – repetition). Supporting references comparing **molecular assays with smear microscopy**  would strengthen this statement.
6. In the **limitations section** , the authors may consider clarifying whether **smear-negative or extrapulmonary samples**  were included, as assay performance can vary in low bacillary load specimens.

==============================

-->Please submit your revised manuscript by Apr 24 2026 11:59PM. If you will need more time than this to complete your revisions, please reply to this message or contact the journal office at plosone@plos.org. When you're ready to submit your revision, log on to https://www.editorialmanager.com/pone/ and select the 'Submissions Needing Revision' folder to locate your manuscript file.

Please include the following items when submitting your revised manuscript:

- A letter that responds to each point raised by the academic editor and reviewer(s). You should upload this letter as a separate file labeled 'Response to Reviewers'.
- A marked-up copy of your manuscript that highlights changes made to the original version. You should upload this as a separate file labeled 'Revised Manuscript with Track Changes'.
- An unmarked version of your revised paper without tracked changes. You should upload this as a separate file labeled 'Manuscript'.

If you would like to make changes to your financial disclosure, please include your updated statement in your cover letter. Guidelines for resubmitting your figure files are available below the reviewer comments at the end of this letter.

-->If applicable, we recommend that you deposit your laboratory protocols in protocols.io to enhance the reproducibility of your results. Protocols.io assigns your protocol its own identifier (DOI) so that it can be cited independently in the future. For instructions see: https://journals.plos.org/plosone/s/submission-guidelines#loc-laboratory-protocols. Additionally, PLOS ONE offers an option for publishing peer-reviewed Lab Protocol articles, which describe protocols hosted on protocols.io. Read more information on sharing protocols at https://plos.org/protocols?utm_medium=editorial-email&utm_source=authorletters&utm_campaign=protocols.

We look forward to receiving your revised manuscript.

Kind regards,

Dr. Minal Dakhave

Academic Editor

PLOS One

**Journal Requirements:**

1. When submitting your revision, we need you to address these additional requirements.

Please ensure that your manuscript meets PLOS ONE's style requirements, including those for file naming. The PLOS ONE style templates can be found at

https://journals.plos.org/plosone/s/file?id=wjVg/PLOSOne_formatting_sample_main_body.pdf and

https://journals.plos.org/plosone/s/file?id=ba62/PLOSOne_formatting_sample_title_authors_affiliations.pdf

2. Please include captions for your Supporting Information files at the end of your manuscript, and update any in-text citations to match accordingly. Please see our Supporting Information guidelines for more information: http://journals.plos.org/plosone/s/supporting-information.

3. If the reviewer comments include a recommendation to cite specific previously published works, please review and evaluate these publications to determine whether they are relevant and should be cited. There is no requirement to cite these works unless the editor has indicated otherwise.

4. Please review your reference list to ensure that it is complete and correct. If you have cited papers that have been retracted, please include the rationale for doing so in the manuscript text, or remove these references and replace them with relevant current references. Any changes to the reference list should be mentioned in the rebuttal letter that accompanies your revised manuscript. If you need to cite a retracted article, indicate the article’s retracted status in the References list and also include a citation and full reference for the retraction notice.

[Note: HTML markup is below. Please do not edit.]

**Reviewers' comments:**

Reviewer's Responses to Questions

**Comments to the Author**

1. Is the manuscript technically sound, and do the data support the conclusions?

The manuscript must describe a technically sound piece of scientific research with data that supports the conclusions. Experiments must have been conducted rigorously, with appropriate controls, replication, and sample sizes. The conclusions must be drawn appropriately based on the data presented.

Reviewer #1: Yes

Reviewer #2: Yes

**********

2. Has the statistical analysis been performed appropriately and rigorously?

Reviewer #1: Yes

Reviewer #2: Yes

**********

3. Have the authors made all data underlying the findings in their manuscript fully available?

The PLOS Data policy requires authors to make all data underlying the findings described in their manuscript fully available without restriction, with rare exception (please refer to the Data Availability Statement in the manuscript PDF file). The data should be provided as part of the manuscript or its supporting information, or deposited to a public repository. For example, in addition to summary statistics, the data points behind means, medians and variance measures should be available. If there are restrictions on publicly sharing data—e.g. participant privacy or use of data from a third party—those must be specified.

Reviewer #1: Yes

Reviewer #2: Yes

**********

4. Is the manuscript presented in an intelligible fashion and written in standard English?

PLOS ONE does not copyedit accepted manuscripts, so the language in submitted articles must be clear, correct, and unambiguous. Any typographical or grammatical errors should be corrected at revision, so please note any specific errors here.

Reviewer #1: Yes

Reviewer #2: Yes

**********

5. Review Comments to the Author

Please use the space provided to explain your answers to the questions above. You may also include additional comments for the author, including concerns about dual publication, research ethics, or publication ethics. (Please upload your review as an attachment if it exceeds 20,000 characters)

**Reviewer #1:**  I have reviewed the manuscript and made the comments below;

1) The author needs to define feasibility from the beginning. Which parameters were used to measure feasibility?

2) L52-54: This is discussing results, it should be reserved to the conclusion section

3) L57: There are no results for smear microscopy, yet it makes it to the conclusion section

4) L78: please use 2025 WHO TB report for 2024 data

5) 106: The prospective nature of the study design, is not clearly spelt out: how it will be done and for which part of the design

6) Results L147-149: This is repeating the methods under results section. Please revise to reduce on the wording.

7) Was smear microscopy using ZN or fluorescent Microscopy? please clarify

8) L166-167: The sensitivity for DST using phenotypic DST as reference had few samples, why not use agreement instead of sensitivity since this has less power as depicted by the wider confidence intervals. Alternatively compare agreement for DST between TrueNAT and GeneXpert and use phenotypic DST as a tie-breaker.

9) please review the PPV for MTB plus vs Microscopy: I get 53.5% instead of 42.4%

10) MTB plus vs Culture and MTB-RIF Vs pDST has been done before, what is new that this data is providing? This manuscript is focused on Mtbplus vs Xpert which should be the main focus. The same with Table 3.

11) Errors and invalids should indicate that it is comparing MTBplus and Xpert in the sub title

12) L201-202: please report in terms of the reduction in invalid results

13) The Rif indeterminate for TRACE call under Xpert, is expected as per the technology, why not present it separate without comparison? Compare Inditerminate and errors for RIF between platforms without TRACE call results.

14) Table 4 (which is referred as Table 3): MTB invalid, indeterminate and RIF indeterminate, ALL contribute to cost and must be discussed. They also require repeat testing and should be discussed under cost and feasibility section.

**Reviewer #2:**  This is an automated report for PONE-D-26-00956. This report was solicited by the PLOS One editorial team and provided by ScreenIT.

ScreenIT is an independent group of scientists developing automated tools that analyze academic papers. A set of automated tools screened your submitted manuscript and provided the report below. Each tool was created by your academic colleagues with the goal of helping authors. The tools look for factors that are important for transparency, rigor and reproducibility, and we hope that the report might help you to improve reporting in your manuscript. Within the report you will find links to more information about the items that the tools check. These links include helpful papers, websites, or videos that explain why the item is important. While our screening tools aim to improve and maintain quality standards they may, on occasion, miss nuances specific to your study type or flag something incorrectly. Each tool has limitations that are described on the ScreenIT website. The tools screen the main file for the paper; they are not able to screen supplements stored in separate files. Please note that the Academic Editor had access to these comments while making a decision on your manuscript. The Academic Editor may ask that issues flagged in this report be addressed. If you would like to provide feedback on the ScreenIT tool, please email the team at ScreenIt@bih-charite.de. If you have questions or concerns about the review process, please contact the PLOS One office at plosone@plos.org.

**********

6. PLOS authors have the option to publish the peer review history of their article (what does this mean?). If published, this will include your full peer review and any attached files.

If you choose “no”, your identity will remain anonymous but your review may still be made public.

**Do you want your identity to be public for this peer review?**  For information about this choice, including consent withdrawal, please see our Privacy Policy.

Reviewer #1: **Yes:**  Willy Ssengooba

Reviewer #2: No

**********

[NOTE: If reviewer comments were submitted as an attachment file, they will be attached to this email and accessible via the submission site. Please log into your account, locate the manuscript record, and check for the action link "View Attachments". If this link does not appear, there are no attachment files.]

To ensure your figures meet our technical requirements, please review our figure guidelines: https://journals.plos.org/plosone/s/figures

You may also use PLOS’s free figure tool, NAAS, to help you prepare publication quality figures: https://journals.plos.org/plosone/s/figures#loc-tools-for-figure-preparation.

NAAS will assess whether your figures meet our technical requirements by comparing each figure against our figure specifications.

Attachment Submitted filename: report_10.64898+2026.01.19.26344389.pdf

# Author comment: Author response to Decision Letter 1

**DOI:** 10.1371/journal.pone.0341988.r002

1 Apr 2026

Academic Reviewer

Comment 1: The introduction could be expanded with additional background on molecular diagnostics for TB and supported with relevant references.

Response: We thank the reviewer for this suggestion. Additional background on molecular diagnostics for tuberculosis has been incorporated into the Introduction section to better contextualize the role of rapid molecular assays in TB diagnosis.

Comment 2: Please provide the manufacturer details and catalog numbers of the kits used for both assays.

Response: We thank the reviewer for this suggestion. Manufacturer details and catalog numbers for both Xpert MTB/RIF Ultra and Truenat MTB Plus/MTB-RIF assays have been added in the Methods (Procedures) section.

Comment 3: The rifampicin resistance targets for the TruPCR assay are not described in the introduction and should be included.

Response: We thank the reviewer for this observation. The targets for rifampicin resistance detection in the Truenat MTB-RIF assay have now been described in the Introduction section.

Comment 4: Additional methodological clarity on both platforms would be helpful. Please clarify whether the assay follows a two-step approach and gene targets.

Response: We thank the reviewer for this valuable suggestion. The Methods section has been revised to provide additional clarity on the workflow of both platforms. The sequential two-step process for Truenat (MTB detection followed by MTB-RIF testing) and the single-step integrated workflow for Xpert MTB/RIF Ultra have been clearly described.

Comment 5: Minor editorial correction required in line 235 (‘TB diagnosis of TB’ – repetition). Supporting references comparing molecular assays with smear microscopy would strengthen this statement.

Response: We thank the reviewer for identifying this error. The typographical repetition has been corrected. Additionally, the statement has been strengthened by including supporting literature highlighting the superior sensitivity of molecular assays compared to smear microscopy, particularly in smear-negative cases.

Comment 6: In the limitations section, clarify whether smear-negative or extrapulmonary samples were included

Response: We thank the reviewer for this important suggestion. The limitations section has been revised to clarify that only pulmonary TB cases were included. It has also been specified that, although both smear-positive and smear-negative cases were included, subgroup analysis based on smear status was not performed.

Reviewer# 1

Comment1: The author needs to define feasibility from the beginning. Which parameters were used to measure feasibility?

Response: We thank the reviewer for this suggestion. Operational feasibility has now been clearly defined in the Introduction section, including the parameters used for its assessment.

Comment 2: L52-54: This is discussing results, it should be reserved to the conclusion section

Response: We thank the reviewer for this observation. Result statements have been retained in the Results section in a descriptive manner, and interpretative statements have been moved

Comment 3: L57: There are no results for smear microscopy, yet it makes it to the conclusion section

Response: We thank the reviewer for this observation. The comparison with smear microscopy has been removed from the conclusion section of the abstract to ensure consistency with the results presented.

Comment 4: L78: please use 2025 WHO TB report for 2024 data

Response: We thank the reviewer for this suggestion. The epidemiological data in the Introduction have been updated in accordance with the latest WHO Global Tuberculosis Report 2025, reflecting the most recent global TB burden estimates.

Comment 5: 106: The prospective nature of the study design, is not clearly spelt out: how it will be done and for which part of the design

Response: The study design has been clarified as a prospective cross-sectional diagnostic accuracy study, and details regarding consecutive enrollment and real-time sample processing have been explicitly described.

Comment 6: Results L147-149: This is repeating the methods under results section. Please revise to reduce on the wording.

Response: We thank the reviewer for this observation. The Results section has been revised to remove redundant methodological descriptions and streamline the wording to present only key findings.

Comment 7: Was smear microscopy using ZN or fluorescent Microscopy? please clarify

Response: We thank the reviewer for this suggestion. The methods used for smear microscopy and mycobacterial culture have now been clearly specified in the Methods section, including the use of fluorescence microscopy (Auramine-O staining) and the BACTEC MGIT 960 system for culture.

Comment 8: L166-167: The sensitivity for DST using phenotypic DST as reference had few samples, why not use agreement instead of sensitivity since this has less power as depicted by the wider confidence intervals. Alternatively compare agreement for DST between TrueNAT and GeneXpert and use phenotypic DST as a tie-breaker.

Response: We thank the reviewer for this insightful suggestion. We acknowledge that the limited number of rifampicin-resistant cases affects the precision of sensitivity estimates, as reflected by wider confidence intervals. While agreement-based analysis was considered, sensitivity and specificity measures were retained to ensure consistency with standard diagnostic accuracy reporting. This limitation has now been explicitly discussed in the manuscript.

Comment 9: Please review the PPV for MTB plus vs Microscopy: I get 53.5% instead of 42.4%

Response: We thank the reviewer for carefully examining the data. We have rechecked the PPV calculation for MTB Plus versus microscopy. Based on the values provided in Table 2 (TP = 61, FP = 83), the PPV is 61/(61+83) = 42.4%, which is correctly reported in the manuscript. We have reconfirmed that all values in the table are internally consistent.

Comment 10: MTB plus vs Culture and MTB-RIF Vs pDST has been done before, what is new that this data is providing? This manuscript is focused on Mtbplus vs Xpert which should be the main focus. The same with Table 3.

Response: We thank the reviewer for this important observation. The Discussion has been revised to emphasize the head-to-head comparison between Truenat MTB Plus and Xpert MTB/RIF Ultra as the primary focus of the study. This has been explicitly highlighted in the opening paragraph of the Discussion.

Comment 11: Errors and invalids should indicate that it is comparing MTBplus and Xpert in the sub title

Response: We thank the reviewer for this suggestion. The subheading has been revised to clearly indicate that the comparison is between Truenat MTB Plus and Xpert MTB/RIF Ultra.

Comment 12: L201-202: please report in terms of the reduction in invalid results

Response: We thank the reviewer for this suggestion. The reduction in invalid results following repeat testing has now been explicitly quantified in the Results section.

Comment 13: The Rif indeterminate for TRACE call under Xpert, is expected as per the technology, why not present it separate without comparison? Compare Inditerminate and errors for RIF between platforms without TRACE call results.

Response: We thank the reviewer for this important observation. Indeterminate rifampicin resistance results in Xpert MTB/RIF Ultra were found to occur exclusively in trace-positive samples, which are an inherent, algorithm-defined feature of the assay. These have now been presented separately and excluded from direct comparison with Truenat MTB-RIF. The Results, Discussion, and corresponding table have been revised accordingly for clarity.

Comment 14: Table 4 (which is referred as Table 3): MTB invalid, indeterminate and RIF indeterminate, ALL contribute to cost and must be discussed. They also require repeat testing and should be discussed under cost and feasibility section.

Response: We thank the reviewer for this important observation. The Discussion section has been revised to include the impact of MTB invalid, indeterminate, and rifampicin indeterminate results on cost and operational feasibility, including the need for repeat testing and increased resource utilization.

Attachment Submitted filename: Response to Reviewers.docx

# Editor report: Decision Letter 1

**DOI:** 10.1371/journal.pone.0341988.r003

**Authors:** Minal Dakhave, Minal Dakhave

11 Jun 2026

Diagnostic accuracy, operational feasibility, and cost considerations of Truenat MTB Plus and Xpert MTB/RIF Ultra for pulmonary tuberculosis

PONE-D-26-00956R1

Dear Dr. Zeeshan Sidiq,

We’re pleased to inform you that your manuscript has been judged scientifically suitable for publication and will be formally accepted for publication once it meets all outstanding technical requirements.

Within one week, you’ll receive an e-mail detailing the required amendments. When these have been addressed, you’ll receive a formal acceptance letter and your manuscript will be scheduled for publication.

An invoice will be generated when your article is formally accepted. Please note, if your institution has a publishing partnership with PLOS and your article meets the relevant criteria, all or part of your publication costs will be covered. Please make sure your user information is up-to-date by logging into Editorial Manager at Editorial Manager® and clicking the ‘Update My Information' link at the top of the page. For questions related to billing, please contact billing support.

If your institution or institutions have a press office, please notify them about your upcoming paper to help maximize its impact. If they’ll be preparing press materials, please inform our press team as soon as possible -- no later than 48 hours after receiving the formal acceptance. Your manuscript will remain under strict press embargo until 2 pm Eastern Time on the date of publication. For more information, please contact onepress@plos.org.

Kind regards,

Dr. Minal. Dakhave

Academic Editor

PLOS One

# Editor report: Acceptance letter

**DOI:** 10.1371/journal.pone.0341988.r004

**Authors:** Minal Dakhave, Minal Dakhave

PONE-D-26-00956R1

PLOS One

Dear Dr. Sidiq,

I'm pleased to inform you that your manuscript has been deemed suitable for publication in PLOS One. Congratulations! Your manuscript is now being handed over to our production team.

At this stage, our production department will prepare your paper for publication. This includes ensuring the following:

* All references, tables, and figures are properly cited

* All relevant supporting information is included in the manuscript submission,

* There are no issues that prevent the paper from being properly typeset

You will receive further instructions from the production team, including instructions on how to review your proof when it is ready. Please keep in mind that we are working through a large volume of accepted articles, so please give us a few days to review your paper and let you know the next and final steps.

Lastly, if your institution or institutions have a press office, please let them know about your upcoming paper now to help maximize its impact. If they'll be preparing press materials, please inform our press team within the next 48 hours. Your manuscript will remain under strict press embargo until 2 pm Eastern Time on the date of publication. For more information, please contact onepress@plos.org.

You will receive an invoice from PLOS for your publication fee after your manuscript has reached the completed accept phase. If you receive an email requesting payment before acceptance or for any other service, this may be a phishing scheme. Learn how to identify phishing emails and protect your accounts at https://explore.plos.org/phishing.

If we can help with anything else, please email us at customercare@plos.org.

Thank you for submitting your work to PLOS ONE and supporting open access.

Kind regards,

PLOS ONE Editorial Office Staff

on behalf of

Dr. Minal Dakhave

Academic Editor

PLOS One
